# The influence of mini-fragment plates on the mechanical properties of long-bone plate fixation

**DOI:** 10.1097/OI9.0000000000000034

**Published:** 2019-04-23

**Authors:** Riley Knox, Patrick Curran, Safa Herfat, Utku Kandemir, Meir Marmor

**Affiliations:** Orthopaedic Trauma Institute, Department of Orthopaedic Surgery, University of California, San Francisco, San Francisco, CA

**Keywords:** biomechanics, fixation, fracture, mechanical testing, mini-fragment plate, orthopaedic surgery, research

## Abstract

**Objective:**

Mini-fragment plates (MFPs) are increasingly used in fracture surgery to provide provisional fixation. After definitive fixation, the surgeon decides whether to remove the plates or leave them in place as additional fixation, based on the perceived biomechanical influence of the MFP. However, there are no current biomechanical studies to guide this decision. Therefore, the purpose of this study was to evaluate the influence of MFPs on the four-point bending and torsional stiffness of long bone transverse and simple wedge fracture fixation constructs.

**Methods:**

Fourth-generation composite bone cylinders were cut to produce transverse (AO-OTA classification 12-A3) and simple wedge (AO-OTA classification 12-B2) fracture models. The specimens were fixed using a low-contact dynamic compression plate (LC-DCP) and MFPs. Specimens were tested in four-point bending and torsion utilizing 3 different MFP orientations.

**Results:**

No statistically significant differences in bending stiffness were found between control and MFP groups for *transverse* fracture constructs. MFPs significantly increased the bending stiffness for *wedge* fracture constructs under certain loading conditions. This increase was observed when MFPs were positioned both orthogonal (85.1% increase, *P = *.034) and opposite (848.2% increase, *P < *.001) to the LC-DCP. MFPs significantly increased the torsional stiffness for both transverse and wedge fracture constructs when MFPs were positioned both orthogonal (transverse: 27.7% increase, wedge: 16.7% increase) and opposite (transverse: 28.4%, wedge: 24.2% increase) to the LC-DCP.

**Conclusions:**

Our results indicate that including MFPs in definitive fixation can increase the bending and torsional stiffness of a long-bone fracture fixation construct. This suggests that the biomechanical influence of MFPs should be considered. However, clinical studies will be required to test the applicability of these findings to the clinical setting.

## 1 Introduction

**Figure 1:**
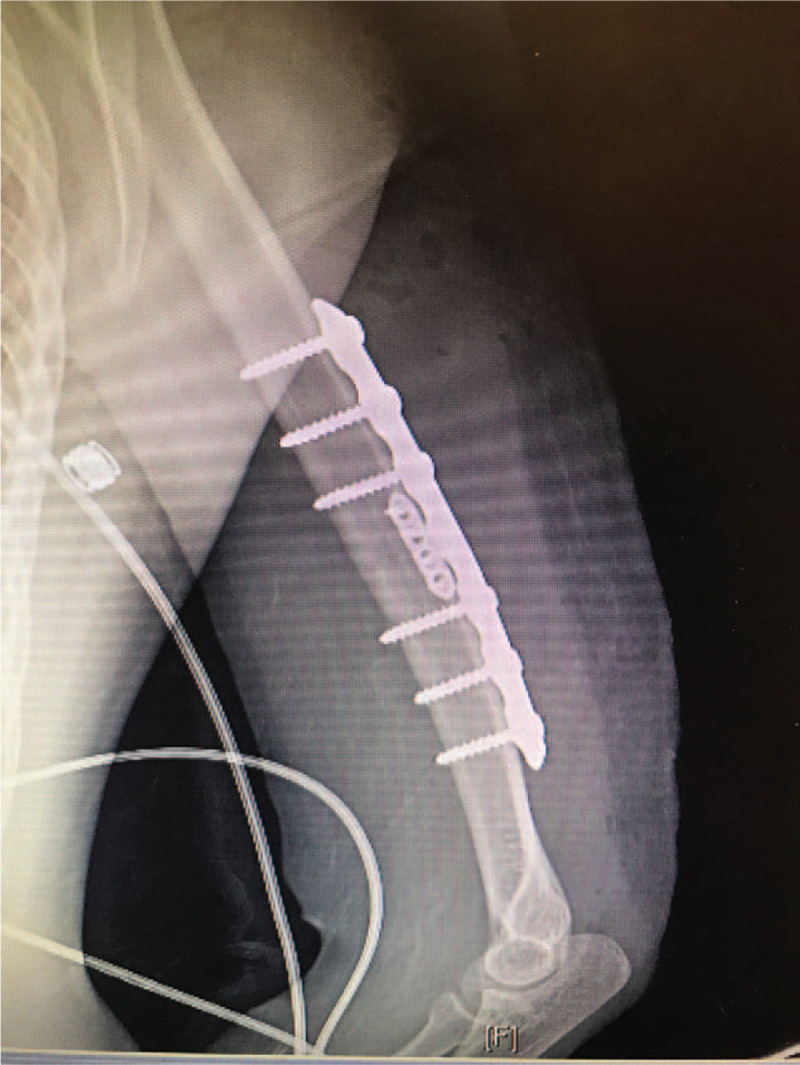
Definitive humeral diaphysis fixation using MFP in conjunction with large plate.

MFPs were originally designed for fixation of small bones or fracture fragments that are too small to be fixed by standard size implants. The ability of these plates to provide provisional fixation for larger fragments prior to applying definitive fixation by larger implants has long been recognized.^[1–3]^ Once applied provisionally, however, the option of leaving them in place after the application of definitive fixation is controversial (Fig. 1). The perceived contribution of these plates to the biomechanical properties of the fixation construct often influences the decision to keep or remove the plates. However, there are no published data on the biomechanical effect that these plates have on the fixation construct. This information may be significant to the surgeon when deciding on the desired stability for a fixation construct.

The purpose of this study was to evaluate the influence of MFPs on the four-point bending and torsional stiffness of long bone transverse and simple wedge fracture fixation constructs. We hypothesized that adding 2.0 mm MFPs in any position would not significantly alter the four-point bending and torsional stiffness of a 4.5 mm LC-DCP definitive fixation construct in long bone transverse and simple wedge fractures.

## 2 Methods

### 2.1 Specimen preparation

**Figure 2:**
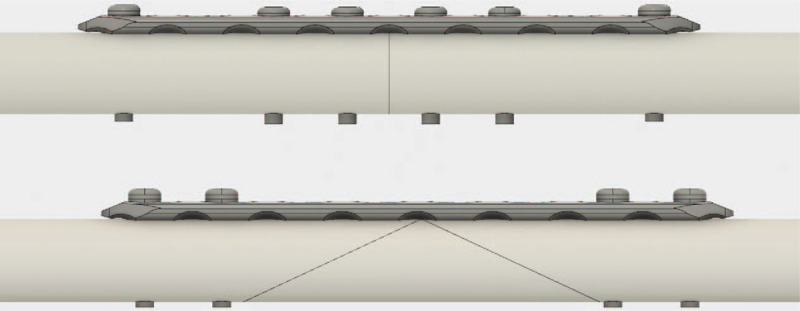
Fracture patterns and screw fixation for transverse (top) and wedge (bottom) fracture models.

**Figure 3:**
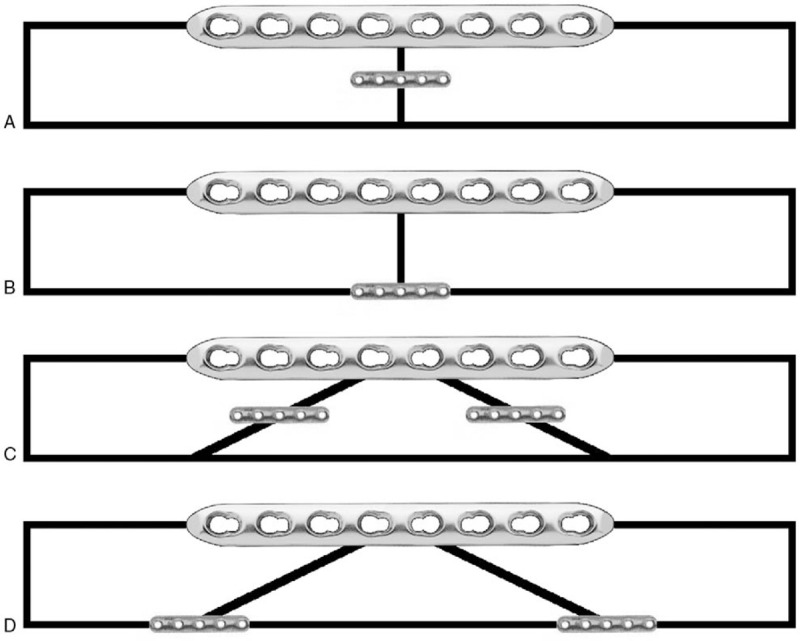
All MFP fixation constructs investigated in this study. Construct (A) transverse fracture, orthogonal miniplate. Construct (B) transverse fracture, opposite miniplate. Construct (C) wedge fracture, orthogonal miniplates. Construct (D) wedge fracture, opposite miniplates.

Two models simulating humeral shaft fractures (transverse group: AO-OTA classification 12-A3; wedge group: AO-OTA classification 12-B2) and fixation constructs were chosen. Ten 250 mm length composite biomechanical grade bone cylinders (#3403-34-1 4th generation composite, 20 mm outer diameter, 3.6 mm wall thickness, 17# solid foam filling; Sawbones, Pacific Research, Vashon, Washington) were cut using a bandsaw to produce 5 transverse fracture specimens and 5 wedge fracture specimens. Specimens were bisected at the longitudinal midpoint to create transverse fractures. Specimens simulating wedge fractures were cut with the wedge vertex aligned with the longitudinal midpoint, with fracture lines aligned at 20° to the longitudinal axis. All specimens were fixed with an 8-hole 4.5 mm LC-DCP (Synthes, West Chester, Pennsylvania). Self-tapping 4.5 mm cortex screws (Synthes, West Chester, Pennsylvania) were used to secure the LC-DCP. Screws were installed bicortically. Transverse fracture specimens were fixed with screws in LC-DCP holes 1, 3, 4, 5, 6, and 8. Wedge fracture specimens were fixed with screws in LC-DCP holes 1, 2, 7, and 8 (Fig. 2). Wedge fracture control models were tested without the wedge fragment due to a lack of mechanical support. Following initial control testing described below, all specimens were fixed with 1 (transverse fracture model) or 2 (wedge fracture model) 5-hole 2.0 mm MFPs (Synthes, West Chester, Pennsylvania) aligned parallel to the longitudinal bone axis. Self-tapping 2.0 mm cortex screws (Synthes, West Chester, Pennsylvania) were used to secure the MFPs. MFP screws were installed unicortically. In addition to the control groups, a total of 4 different fixation constructs were created for mechanical testing (Fig. 3). Each control group, as well as all fixation construct subgroups, contained 5 test specimens.

### 2.2 Mechanical testing setup

**Figure 4:**
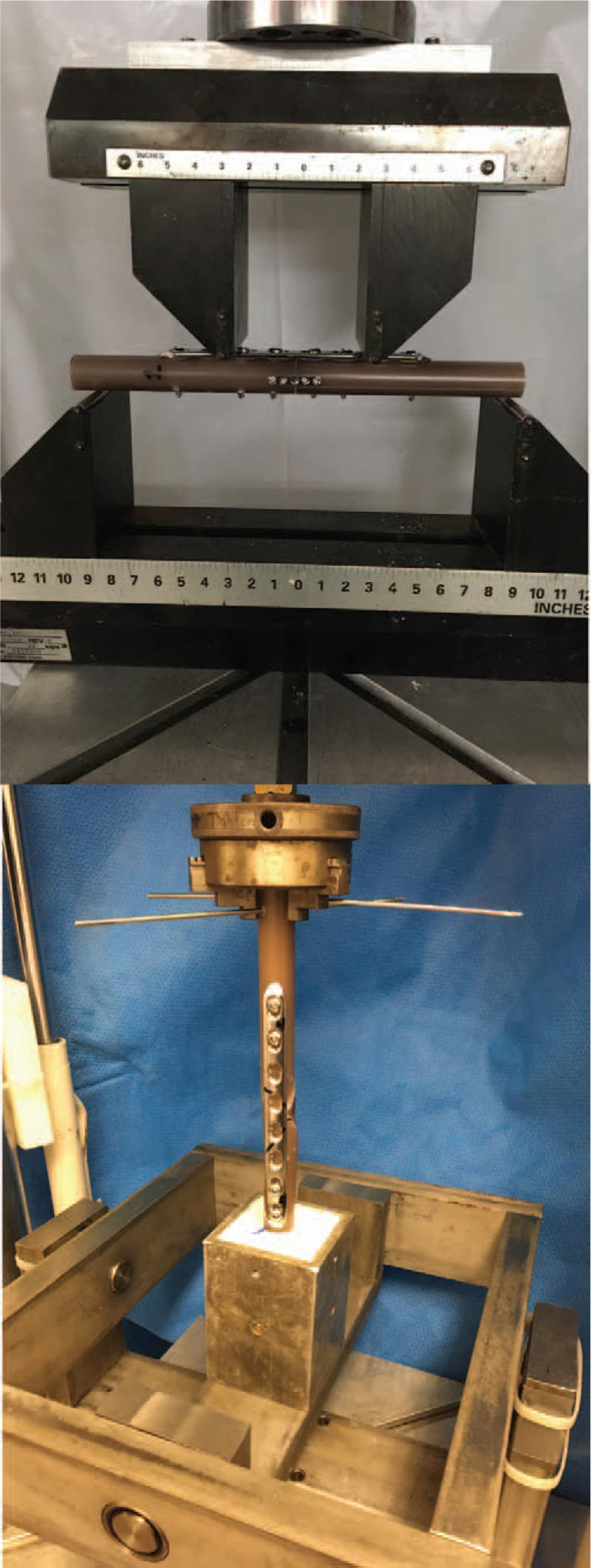
Four-point bending (top) and torsion (bottom) mechanical testing setups.

Biomechanical testing was performed using a servohydraulic material testing machine (Bionix 370 Axial/Torsional, MTS Systems Corp, Eden Prairie, Minneapolis) equipped with a 6-degree-of-freedom load cell (MC5-2500, AMTI, Berkshire, England). Each sample was subjected to four-point bend loading using standard 4-point bending fixtures (MTS Systems Corp, Eden Prairie, Minneapolis) with 76.2 mm distance between the top fulcrums and 228.6 mm distance between the bottom fulcrums. Specimens were positioned with the longitudinal midpoint centered between the force applicators (Fig. 4). Specimens were not fixed to the bottom fulcrums; however, the applied forces produced sufficient friction to prevent specimens from rolling.

Before torsional testing, 1 end of each specimen was embedded in potting resin (Smooth-Cast 300, Smooth-On Inc., East Texas, Pennsylvania) to a depth of 45 mm and a distance of 5 mm from the end of the LC-DCP. The potting fixture was secured to the MTS load cell using a custom jig that allowed for unconstrained rotation in the 2 bending planes. The free end of the specimen was then secured to the machine actuator using a 3-jaw lathe chuck. Two Kirschner wires were drilled through this end to prevent rotation of the sample in the chuck while applying torque (Fig. 4).

### 2.3 Loading protocol

All specimens were tested in four-point bending and torsion utilizing 3 MFP configurations. The different configurations were tested in the following order:

1. No MFPs present (control).
2. MFPs positioned 90° from the LC-DCP around the specimen longitudinal axis (orthogonal).
3. MFPs positioned 180° from the LC-DCP around the specimen longitudinal axis (opposite).

All specimens were tested according to the following 2 loading protocols:

1. Cyclic four-point bending stiffness test: 50–200 N applied at 1 Hz for 20 cycles. Specimens were tested in 4 different loading orientations: loading directly on the LC-DCP, loading directly opposite the LC-DCP, and loading at both 90° orientations to the LC-DCP.
2. Cyclic torsional stiffness test: 0.5–1.5 N m applied at 1 Hz for 20 cycles.

All tests were performed in the same order on all specimens. Four-point bending tests were performed first in the following order: loading directly on the LC-DCP, loading directly opposite the LC-DCP, loading at 90° to the LC-DCP on the side where the MFP was installed (regardless of its orientation), loading at 90° to the LC-DCP opposite to the MFP installation side. Torsional tests were performed once all four-point bending tests had been performed for all MFP orientations. In total, 5 tests (4 bending, 1 torsion) were performed per specimen in each of the 3 MFP configurations.

The cyclic peak load was chosen such that applied bending and torsional moments were consistent with those observed during daily activities.^[4]^ Additionally, a preliminary specimen was tested to ensure cyclic loading was performed in the specimens’ elastic region, avoiding plastic (or permanent) deformation.

For both bending and torsional tests, the specimen was preloaded to the minimum cyclic load and allowed to settle for 30 seconds. Cyclic testing was initiated once the preload settled at the minimum cyclic load.

### 2.4 Data acquisition and statistical analysis

Vertical displacement and applied compressive force were recorded during four-point bend testing. Rotational angle and torque were recorded during torsional testing. All data were recorded by the MTS controller at 102.4 Hz. Four-point bending and torsional stiffness values were calculated from the second-to-last cycle of each test. For specimens where settling effects were present during the loading cycle, the stiffness value was calculated from the later, settled portion of the force–displacement curve. To ensure no permanent damage was done to either the cylinders or the implants, *R*-squared values were calculated for all loading curves. Prior to performing statistical comparisons, normality of the data was confirmed using the Shapiro–Wilk test. Repeated measures analysis of variance (ANOVA) was used to identify loading conditions where statistically significant differences between groups may be present. Paired, 2-tailed *t*-tests (*P < *.05) were then used to compare bending and torsional stiffness between the control group and each MFP configuration, as well as between MFP configurations, in groups where ANOVA indicated significant differences were present. Finally, post-hoc power analysis was performed.

## 3 Results

### 3.1 Transverse fracture model

Adding an MFP in any orientation did not affect the four-point bending stiffness for any loading condition. Adding a MFP significantly increased torsional stiffness in both groups (orthogonal: 27.7% increase in mean, *P = *.001; opposite: 28.4% increase in mean, *P = *.02). No significant differences were found in four-point bending or torsional stiffness between the orthogonal and opposite MFP configurations (Table 1). However, due to the small sample size, many of these comparisons were underpowered (power < 80%) as determined from post-hoc power analysis. *R*-squared values (bending, range 0.99–0.9999; torsion, range 0.9782–0.9986) confirm test specimens were not permanently damaged during testing.

**Table 1:**
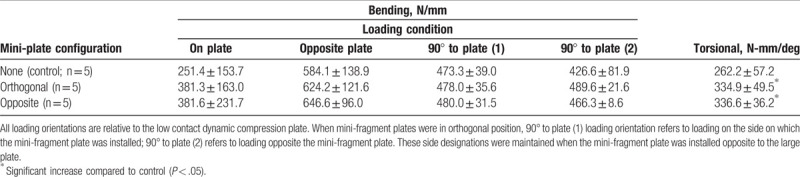
Stiffness values (mean ± standard deviation) for transverse fracture specimens.

### 3.2 Wedge fracture model

Adding MFPs significantly increased the four-point bending stiffness in both groups (orthogonal: 85.1% increase in mean, *P = *.03; opposite: 848.2% increase in mean, *P < *.001) when loading on the LC-DCP. There was no difference in four-point bending stiffness for other loading orientations. Adding MFPs significantly increased torsional stiffness in both groups (orthogonal: 16.7% increase in mean, *P < *.001; opposite: 24.2% increase in mean, *P = *.007). Four-point bend stiffness was significantly greater in the opposite MFP group compared to the orthogonal MFP group when loading directly on (458.2% increase in mean, *P < *.001) or directly opposite to (66.8% increase in mean, *P = *.007) the LC-DCP. There was no significant difference in four-point bending stiffness in either MFP group when loading 90° from the LC-DCP on either side. There was also no significant difference in torsional stiffness between the 2 MFP configurations (Table 2). As in the transverse group, post-hoc power analysis determined many of these comparisons were underpowered (*P* < 80%) due to small sample size. *R*-squared values (bending, range 0.99–0.9996; torsion, range 0.9965–0.9998) confirm test specimens were not permanently damaged during testing.

**Table 2:**
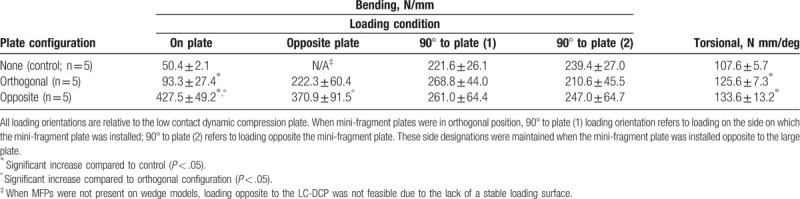
Stiffness values (mean ± standard deviation) for wedge fracture specimens.

## 4 Discussion

The results of this study show that the addition of MFPs to long-bone plate fixation constructs significantly increases bending stiffness in wedge fractures and torsional stiffness in transverse and simple wedge fractures. Thus, our initial hypothesis was rejected. These findings suggest that surgeons may need to consider the biomechanical effect of MFPs when adding them to a definitive fixation construct.

Use of MFPs is part of the armamentarium of the surgeon in long bone fracture fixation and has drawn more attention in recent years.^[5–7]^ While primarily used for provisional fixation, removing or leaving them in place has been a topic of discussion. The knowledge of MFP influence on fixation rigidity will help with the decision to remove or leave them in place, however there is no previous biomechanical study regarding this influence.

From a purely mechanical standpoint, one could expect that the addition of MFPs to a plate fixation construct could increase the bending stiffness of the construct. In transverse and wedge fracture models, the application of a bending-inducing load will cause the fracture gap to widen, with the widening having greatest magnitude at the aspect farthest from the application surface. Thus, applying an MFP across the fracture gap should, in theory, resist this widening and thereby increase stiffness. It follows that placing an MFP where the widening is greatest, that is, the surface opposite the applied load, would cause the greatest increase in stiffness. In this study, we did observe a 50% increase in mean bending stiffness in transverse fractures when load was applied to the LC-DCP and the MFP was placed opposite to the plate (Table 1). This increase in mean bending stiffness did not reach statistical significance (*P = *.12); however, this was an underpowered comparison (power = 0.275) and could have benefited from a larger sample size. It may be that, due to the relatively small size of the MFP, adding an MFP does not effectively increase the stiffness of the construct. In other words, the LC-DCP is still responsible for the vast majority of load bearing. Conversely, the MFPs did have a significant impact on torsional stiffness in both transverse and wedge fractures. This is because, in a construct utilizing only an LC-DCP, the LC-DCP alone is not sufficient to anchor the bone fragments around the torsional axis; in general, 2 fixation points are necessary to prevent rotation around an axis. The MFP provides this second fixation point, thus increasing torsional stiffness.

The fracture pattern and the expected type of healing (i.e., primary or secondary) may dictate the use of MFPs as part of the definitive fixation. Primary healing requires rigid fixation over a perfectly reduced fracture gap, while secondary healing may be achieved without perfect reduction or rigid fixation and is in fact enhanced by some axial fragment motion.^[8]^ It follows that surgeons should aim to induce primary healing when they are able to achieve perfect reduction, while allowing fractures that cannot be perfectly reduced to undergo the secondary healing process. To properly induce primary healing, axial strain at the fracture gap must be kept below 2%,^[8]^ necessitating as rigid a fixation as possible. Therefore, in simple patterns, such as the transverse fracture modeled in this study, addition of MFPs may be considered. Conversely, when a perfect reduction is unattainable and secondary healing is expected, MFPs may only be appropriate for provisional reduction. However, because we did not measure interfragmentary motion in this study, we cannot determine if MFPs can be used to induce primary over secondary healing by limiting axial strain to less than 2%. Rather, MFPs may be used to increase fixation rigidity in cases where primary healing can be expected. Use of MFPs may be considered an extra step to ensure primary healing, rather than the catalyst that drives primary instead of secondary healing. However, further studies are needed prior to applying the results of this study in clinical practice.

The use of MFPs in clinical practice has not been associated with complications. Oh et al^[2]^ reported zero nonunion complications in 39 cases across 7 different fracture regions, indicating the use of MFPs is not directly related to the development of nonunion complications. As in our study, this group used 2.0 mm MFPs. However, a key distinction is that this group placed definitive fixation directly on top of the provisionally installed MFP. This means, according to the mechanical theory described above, the MFP is less involved in creating stability in the definitive construct than if it were, for example, positioned opposite to the larger plate. Similarly, Archdeacon and Wyrick^[9]^ reported only a single nonunion among 28 tibial metadiaphysis fracture cases. However, the specific nonunion case was encountered in a high energy, highly comminuted, segmental fracture, indicating fracture complexity was the critical factor in nonunion development. These clinical results suggest that the use of MFPs does not contribute to nonunion.

This study had several limitations inherent to biomechanical studies. First, composite bone models are not a perfect stand-in for cadaveric models or live patients. In addition to lacking the viscoelastic mechanical properties inherent to biological tissues, composite models possess perfectly uniform geometry, which directly influences the construct's mechanical response under all loading modes. On the other hand, standardization of the bone and fracture model, thus avoiding variations such as in a cadaveric model, was critical to identify the specific influence of MFPs. Second, this study only investigated unimodal loading. Activities in vivo rarely, if ever, exert a single type of load (torsion, bending, etc.) on a structure. Therefore, this study did not perfectly mimic in vivo biomechanical loading. Third, the fracture models used in this study were simplified relative to what may be seen in vivo. Each fracture was perfectly cut with a bandsaw, and no additional fragments were present. It is unclear whether the same trends seen in this study would apply to more complicated fracture models, including those with jagged fracture lines and/or necessitating more provisional MFPs, indicating this may be an area for future research. Finally, no load to failure or fatigue testing was performed in this study. MFPs used in this study are much smaller and mechanically weaker than the corresponding LC-DCP, and thus are likely to fail at lower loading magnitudes and/or after a lower number of loading cycles. This creates a potential concern regarding the long-term use of MFPs that were not originally designed for permanent fracture augmentation. The long-term effects of MFPs on fracture fixation performance must be examined in future research. Lastly, the limited sample size resulted in some underpowered comparisons. Despite these limitations, this study provides preliminary biomechanical evidence regarding the influence of MFPs in the context of long bone fixation. However, further biomechanical studies are needed prior to applying biomechanical findings to the clinical setting.

In conclusion, the addition of MFPs to a long bone fixation construct may significantly increase both torsional and bending stiffness, depending on the fracture pattern. The findings of this study suggest that surgeons should consider the biomechanical effects of leaving MFPs as part of their final fixation construct. Future studies are also needed to address the use of MFPs in more complicated fracture types and in clinical practice.
